# Penile mucinous carcinoma: A case report

**DOI:** 10.3892/ol.2014.2839

**Published:** 2014-12-30

**Authors:** HAKAN ÖZTÜRK

**Affiliations:** Department of Urology, School of Medicine, Sifa University, Izmir 35240, Turkey

**Keywords:** penile cancer, mucinous carcinoma, pathology, diagnosis

## Abstract

Penile cancer is an extremely rare form of urological cancer that usually originates in the epithelium of the inner preputium or glans. Major etiological factors include phimosis, poor penile hygiene and smoking. Nearly 95% of penile cancers are squamous cell carcinomas and usually occur in the sixth decade of life. The disease exhibits two variants, namely metastatic and atypical disease. Squamous differentiation may also present with mucinous metaplasia. An extremely limited number of case reports on penile cancer are available in the literature. The present study reports the case of a 39-year-old patient with penile mucinous adenocarcinoma who was admitted with the complaint of perineal discharge, which is, to the best of our knowledge, the first case in the literature. The patient underwent total penectomy and inguinal lymph node dissection. The tumor was staged as T4N1M0, according to the European Association of Urology’s tumor-node-metastasis classification system. The patient was treated with pelvic radiotherapy and six cycles of systemic neoadjuvant chemotherapy with cisplatin and paclitaxel simultaneously, over a period of four months. After nine months of follow-up the patient succumbed to the disease due to widespread metastases.

## Introduction

Penile cancer is an extremely rare form of urological cancer, with an incidence of 0.1–8.3/100,000 males (1,2). The annual incidence is <1/100,000 males in the USA and in European countries (2). However, it is an important global health concern due to the higher incidence, up to 10–20%, in several countries and worldwide (2). The incidence of the disease is highest in Brazil, Uganda and India, while it is lower in the Jewish and Muslim communities, in which infants and children are mostly circumcised. In addition, early circumcision reduces the risk of penile cancer by three to five times (1).

Predisposing factors for penile cancer include chronic inflammatory diseases, such as phimosis, balanoposthitis and balanitis xerotica obliterans, ultraviolet phototherapy, multiple sexual partners, an early age at first intercourse and a previous history of condyloma. Smoking and intercourse with a partner infected with human papilloma virus (HPV) types 6, 11, 16 or 18 are also among the risk factors (3). In addition, the presence of high-risk HPV DNA is critical in the prognosis of the disease, as this may reduce the survival rate (4). Furthermore, defects in tumor suppressor genes, including p53 and Rb, play an important role in the development of cancer (5). As smegma has been reported to be likely to exert carcinogenic effects, circumcision has been considered to reduce the incidence of smegma (6).

Squamous penile cancer accounts for >95% of all malignant penile cancers. Cutaneous horn and Bowenoid papulosis of the penis and balanitis xerotica obliterans, also termed lichen sclerosus, are pre-malignant lesions of penile cancer. Additionally, penile intraepithelial neoplasia, a form of carcinoma *in-situ*, erythroplasia of Queyrat and Bowen’s disease are potential risk factors for penile cancer (3). Nearly 30% of these conditions result in invasive cancer. Patients are usually asymptomatic and exhibit a variable clinical presentation, which may be accompanied by indurated and growing papules, and pustule warts or ulcerative tumors. Early symptoms of the disease are mainly itching and burning in the area of the preputium (3). The present study reports the case of a 39-year-old patient with penile mucinous adenocarcinoma who was admitted with the complaint of perineal discharge.

## Case report

A 39-year-old male patient was admitted to Ataturk Education and Research Hospital (Izmir, Turkey) due to a complaint of perineal discharge. The patient’s medical history revealed that a solid mass had been observed in the perineal area three years prior to the present study and perineal abscess drainage had been performed twice. With an additional complaint of stranguria, the patient also underwent an internal urethrotomy twice for a urethral stricture. A biopsy specimen was obtained from the area in which the abscess drainage was applied one month prior to admission. The medical history also reported that circumcision had been performed at the age of two. The patient possessed poor penile hygiene and a history of smoking.

Systemic examination findings were normal. Urogenital examination revealed a palpable solid flat mass, 8 cm in size, which originated from the left side of the penis glans. The mass involved the urethra in the ventral view of the penis and the spongious body, with an invasion of the left corpus cavernosum, which advanced through the left side of the radix penis and scrotum. A continuous perineal induration and hyperemia were present. An orifice of the urethral fistula in the perineum was observed. A painful lymphadenopathy, 1.5 cm in size, was found in the left superficial inguinal lymph node chain. Digital rectal examination revealed anal edema. Two painless and soft nodules, each 1 cm in size, were observed in the anterior wall of the rectum. The testicles were normal. The biochemical results were as follows: White blood cell count, 6,400/μl (normal range, 4.0–11.0/μl); hemoglobin, 12.9 g/dl (normal range, 12.8–15.5 g/dl); platelet count, 326,000/l (normal range, 150,000–450,000/l); total bilirubin, 0.73 mg/dl (normal range, 0.1–1.2 mg/dl); direct bilirubin, 0.21 mg/dl (normal range, 0.1–0.4 mg/dl); alkaline phosphatase, 46 U/l (normal range, 44–147 U/l); glucose, 86 mg/dl (normal range, 70–101 mg/dl); blood urea nitrogen, 13 mg/dl (normal range, 8–20 mg/dl); creatinine, 1.0 mg/dl (normal range, 0.6–1.2 mg/dl); albumin, 2.7 g/dl (normal range, 3.4–4.7 g/dl); aspartate aminotransferase, 98 U/l (normal range, 10–41 U/l); alanine aminotransferase, 30 U/l (normal range, 10–44 U/l); calcium, 7.9 mg/dl (normal range, 8.5–10.5 mg/dl); sodium, 142 mmol/l (normal range, 134–143 mmol/l); potassium, 3.8 mmol/l (normal range, 3.5–5.5 mmol/l); chlorine, 106 mmol/l (normal range, 98–107 mmol/l); sedimentation rate, 33/h (normal range, <15/h); carcinoembryonic antigen, 4.88 ng/dl (normal range, <7 ng/dl); and prostate-specific antigen, 0.7 ng/ml (normal range, <2.5 ng/ml). An angiogram of the fistula discharge revealed culture positivity for *Klebsiella pneumoniae*. Susceptibility to piperacillin + tazobactam, cefoperazone + sulbactam and meropenem was detected.

**Figure 1:**
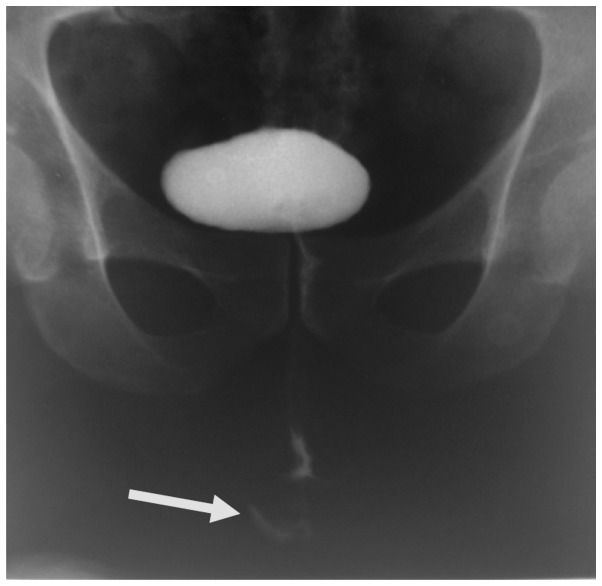
Retrograde urethrography revealing a urethral fistula (arrow).

**Figure 2:**
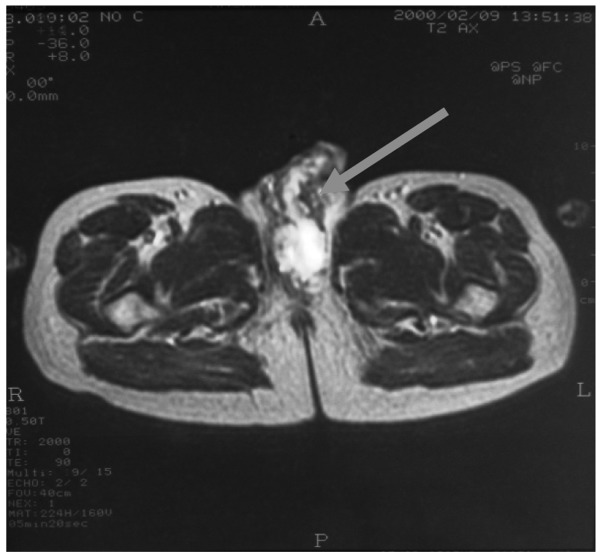
Magnetic resonance imaging revealing a mass in the left cavernous body (arrow).

Abdominal ultrasound and thoracoabdominal computed tomography (CT) produced normal findings. The intravenous pyelography and cystography results were also normal. A fistula at bulbar urethral level was found in the retrograde urethrography (Fig. 1). Rectosigmoidoscopy demonstrated an internal hemorrhoid, while colonoscopy revealed normal findings. Upper gastrointestinal endoscopy revealed antral gastritis. Magnetic resonance imaging (MRI) of the pelvis revealed a heterogeneous mass in the left side of the radix penis and proximal scrotum. Spongious body involvement was evident. Bulbous and cavernous portions of the urethra were infiltrated, whereas the prostate, vesiculae seminalis and testicles were normal (Fig. 2).

Pathological examination of the perineal open biopsy specimens resulted in the diagnosis of mucinous adenocarcinoma. The diagnosis was confirmed by pathological consultation of the same specimens. The patient underwent total penectomy and inguinal lymph node dissection. Intraoperative frozen section analysis revealed a poorly-differentiated mucinous micropapillary tumor originating from the glans to the radix penis, which infiltrated the corpus cavernosum and invaded two-thirds of the penile wall. The tumor was 14×3.5 cm in length with a full-thickness tissue depth of 2.5 cm. One of the inguinal lymph nodes was invaded. However, there was no metastatic disease, as confirmed by positron emission tomography (PET)/CT scans. According to the tumor-node-metastasis staging system, the tumor was staged as T4N1M0.

**Figure 3:**
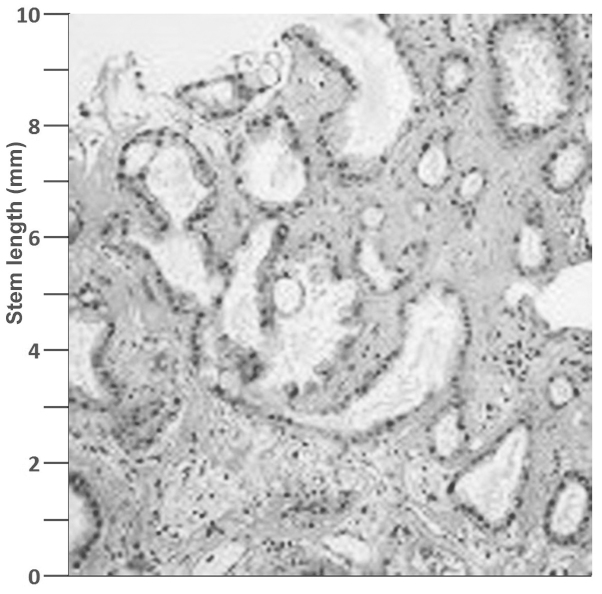
Hematoxylin and eosin staining revealing mucinous islets and tumor cells with alveolar structures, clear cytoplasm and small nucleoli (magnification, ×40).

**Figure 4:**
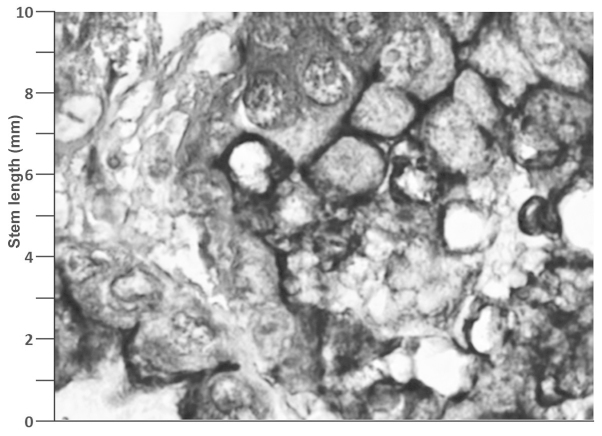
Mucin 1 antibody staining (magnification, ×100).

Intraoperative specimens and perineal specimens, in particular, were analyzed by a pathologist who was experienced in the field of uro-oncology. Hematoxylin and eosin staining revealed mucinous islets (Fig. 3). In addition, large pools of mucin arranged in lobules, separated by collagenous septae were identified. The mucin 1 antibody was labeled in the immunohistochemical examination (Fig. 4), which stained strongly, indicating increased Muc 1 expression.

The patient was treated with pelvic radiotherapy (50 Gy) and systemic neoadjuvant chemotherapy (cisplatin and paclitaxel) simultaneously. After nine months of follow-up the patient succumbed to the disease due to widespread metastases.

Written informed consent was obtained from the patient who participated in the present study. All procedures followed were in accordance with the ethical standards of the Atatürk Education and Research Hospital committee on human experimentation (institutional and national) and with the Helsinki Declaration of 1975, as revised in 2008.

## Discussion

To the best of our knowledge, the present case is the first case of primary mucinous adenocarcinoma of the penis in the literature. Metastatic carcinoma of the penis, one of the rare forms of penile tumors, was excluded by assessing the possible sites of origin by thoracic CT, abdominopelvic MRI and PET/CT. In particular, gastrointestinal tumors are likely to metastasize to the penis, but metastatic carcinoma of the penis may also arise from bladder, prostate and kidney tumors (4,5). In the present patient, endoscopic examination of the gastrointestinal system and biopsy specimens revealed no pathology, with the exception of antral gastritis and hemorrhoids. A mucinous carcinoma originating from the penis glans to the radix penis with inguinal lymph node metastasis suggested primary mucinous adenocarcinoma.

The underlying mechanism and histopathogenesis of mucinous cancer of the penis has yet to be elucidated. Squamous cell carcinoma, the most common form of penile cancer, relies upon the malignant transformation of a pre-malignant lesion (7). These tumors indicate damaged DNA differentiation in the healthy cellular cycle. Possible mechanisms that induce malignant transformation include chronic irritation, smoking and poor penile hygiene. Processes in chronic inflammation may stimulate metaplasia and differentiation. One-third of penile squamous cell carcinoma is associated with the malignant transformation of pre-malignant penile lesions (8). Furthermore, viral infections, including HPV, may cause penile cancer through DNA damage. In addition, defects in tumor suppressor genes, including p53, interrupt the cellular cycle and cause apoptosis (4,5). Loss of such genes contributes to DNA damage and accelerates malignant transformation.

To the best of our knowledge, mucinous adenocarcinoma of the penis has not yet been defined in the literature. However, there have been case studies on mucinous metaplasia, which is considered to be a predisposing factor for penile cancer (9). In addition, a previous study has reported the presence of mucin-producing cells presenting with metaplasia in the preputium in the literature (9). Another study demonstrated a higher incidence of acid-mucin cells in the preputium compared with other areas (10). The underlying mechanism of mucinous transformation remains unknown due to the limited number of studies in the literature. Mucinous metaplasia of the penis is a rare condition with unknown origin (9) that is usually observed in the elderly and has been reported to be associated with chronic balanitis or Zoon’s balanitis (11), which is often overlooked in clinical practice. Only eight cases are available in the literature (9,10,12–14). It is hypothesized that mucinous metaplasia may initially present as a pre-malignant lesion of penile mucinous adenocarcinoma and is associated with malignant transformation due to continuous chronic irritation. However, the exact association between Zoon’s balanitis, mucinous metaplasia and adenocarcinoma remains unknown.

In conclusion, to the best of our knowledge, the present case is the first case of primary mucinous adenocarcinoma of the penis in the literature. The patient was treated with pelvic radiotherapy (50 Gy) and systemic neoadjuvant chemotherapy (cisplatin and paclitaxel) simultaneously. However, after nine months of follow-up the patient succumbed to the disease due to widespread metastases. Although the histopathological mechanism is indefinite, mucinous metaplasia is considered likely to be a risk factor for penile cancer. Therefore, patients with mucinous metaplasia should be closely followed for cancer development.
